# Emergence of spatio-temporal variations in chemotherapeutic drug efficacy: in-vitro and in-Silico 3D tumour spheroid studies

**DOI:** 10.1186/s12885-020-07677-5

**Published:** 2020-12-07

**Authors:** M. V. Sheraton, G. G. Y. Chiew, V. Melnikov, E. Y. Tan, K. Q. Luo, N. Verma, P. M. A. Sloot

**Affiliations:** 1 HEALTHTECH NTU, Interdisciplinary Graduate School, Nanyang Technological University, Singapore, Singapore; 2 Complexity Institute, Nanyang Technological University, Singapore, Singapore; 3 School of Chemical and Biomedical Engineering, Nanyang Technological University, Singapore, Singapore; 4 Department of General Surgery, Tan Tock Seng Hospital, Singapore, Singapore; 5 Faculty of Health Sciences, University of Macau, Taipa, Macau, China; 6 Department of Chemical Engineering, Indian Institute of Technology Kanpur, Kanpur, India; 7 ITMO University St. Petersburg, Russian Federation, St Petersburg, Russia; 8 Institute for Advanced Study, University of Amsterdam, Amsterdam, The Netherlands

**Keywords:** Reaction-diffusion model, Pharmacokinetics, Pharmacodynamics, Mitotic spindle stabilization, Cytotoxicity

## Abstract

**Background:**

The mechanisms of action and efficacy of cisplatin and paclitaxel at cell population level are well studied and documented, however the localized spatio-temporal effects of the drugs are less well understood. We explore the emergence of spatially preferential drug efficacy resulting from variations in mechanisms of cell-drug interactions.

**Methods:**

3D spheroids of HeLa-C3 cells were treated with drugs, cisplatin and paclitaxel. This was followed by sectioning and staining of the spheroids to track the spatio-temporal apoptotic effects of the drugs. A mechanistic drug-cell interaction model was developed and simulated to analyse the localized efficacy of these drugs.

**Results:**

The outcomes of drug actions on a local cell population was dependant on the interactions between cell repair probability, intracellular drug concentration and cell’s mitosis phase. In spheroids treated with cisplatin, drug induced apoptosis is found to be scattered throughout the volume of the spheroids. In contrast, effect of paclitaxel is found to be preferentially localized along the periphery of the spheroids. Combinatorial treatments of cisplatin and paclitaxel result in varying levels of cell apoptosis based on the scheduling strategy.

**Conclusions:**

The preferential action of paclitaxel can be attributed to the cell characteristics of the peripheral population. The model simulations and experimental data show that treatments initiated with paclitaxel are more efficacious due to the cascading of spatial effects of the drugs.

## Background

Multicellular tumour spheroid models have become an invaluable in-vitro method for gaining insight into the interaction between tumour cells and their microenvironment. 3D spheroid models have been able to replicate the features of in-vivo tumour, such as activation of pathways upon cell-cell interactions [1], presence of concentration gradients within the tumour, and heterogenous microenvironment which can alter pharmacokinetics (PK) of the chemotherapeutic drug [2]. These systems provide a clear advantage over the classical monolayer or 2D culture systems which do not resemble the in-vivo conditions of tumour growth. Moreover, 2D cultures exhibit exponential cell proliferation and altered cell morphologies, thus preventing them from becoming reliable drug testing setup. Co-culture 3D spheroids can capture the cell-cell interactions, elevated levels of protein expression and physiological conditions [3, 4] present in-vivo, thus presenting themselves as a suitable candidate for drug screening and post-screening analysis.

Patra et al. [5] designed a high throughput microfluidic device capable of forming multiple (5000 numbers) uniformly sized spheroids in a semi-autonomous fashion. By combining the device with flow cytometry, it was possible to conduct drug testing and analysis on the formed 3D spheroids. Such devices could expedite drug screening and be a useful tool for tissue in chip studies. Sectioning of a drug treated spheroid could reveal the internal architecture [6] and changes in morphology of the cells due to drug effects. This could also aid in understanding the PK of the drugs and its effects on cell cytology. However, sectioning and staining of spheroids is a time-consuming process. There are methods [7, 8] proposed in literature, which help to overcome such tedious process. Anand et al. [7] proposed a real-time FRET-based cell viability monitoring system for detecting apoptosis in a 3D spheroid. Using confocal microscopy, it should be possible to observe in real time the cells in the different stages of their life cycles at different layers inside the spheroid.

There exists a few concerns regarding, the use of 3D spheroid models for therapeutic screening: (1) size reproducibility of the spheroids, (2) factors (cell density, extracellular matrix) that affect mass transport of drugs, and (3) time spent in single sample testing or analysis. It has been reported that small variations in spheroid sizes could lead to large variations in the sensitivity of tumour cells to photodynamic treatment [9] and anti-cancer drug treatment [10] . West et al. [9] showed that the treatment sensitivity of the cells decreased by approximately 12 folds with a five-fold increase in spheroid size. This clearly shows that characteristics of spheroid systems are not scalable or directly extendable from a system of different sizes. Spheroids of larger sizes have also been shown [10] to be resistant to drug treatment. Therefore, if a drug test were to be carried out on a spheroid and proven successful, it does not necessarily imply that it works on a larger in-vivo tumour at the same dosage. In the same study, it was also observed that large spheroids were populated with more apoptotic cells than viable cells. This observation indicates that there are variations in the spatial distributions of different cell types and cell packing density within the spheroid. These variations could alter the drug diffusion kinetics and the uptake rate at different layers within the spheroid leading to a localized effect, which would otherwise be absent in the homogenously packed small-sized spheroids. Further, spheroid systems that use alginate or agarose coatings [11–13] could lead to changes in transport phenomena and cytotoxicity within the spheroid due to imperfections in coating or non-uniform drying of the agarose material.

Spheroid models for drug screening are known to be tedious and time-consuming. Friedrich et al. [14] proposed that the time taken to run the complete protocol for a proper spheroid-based drug screening will be around 170 h. The post screening analysis must be carried out for the next 14 days in succession. Also, analysis must be carried out for different drug dosages, spheroids of different sizes, supporting material of different stiffness, and combinations of all the above in case of combinatorial drug tests. It is well known that drug concentration is not the only free parameter in the spheroid model, factors such as mass transport within the tumour, size of the tumour and local density of viable cells, can also influence the drug efficacy. Because of this inherent complexity, it is hard to separate and study the major contributors of drug efficacy and their impacts on the tumour environment through a single experiment or multiple experiments. In some cases, it is not even possible to tailor the experimental setups needed for drug testing with adequate accuracy, using existing 3D spheroid culture techniques, such as setups needed for studying the effects of variation in intra-tumoral mass transfer on the drug efficacy. One way to handle such complexities associated with interactions of the cells, variations in local densities and drug’s mass transfer is through the use of *in-silico* models. If sufficient data on diffusion parameters, lethal drug concentrations and drug mechanisms are available, all major factors controlling tumour-drug evolution can be included into a computational model and simulated to predict the outcome of drug screening experiments,

Cellular automata (CA)-based models have been used to simulate 3D tumour growth [15, 16]. These models include the interaction between the tumour cells and their micro-environment. Alarcon et al. [16] proposed a cellular automata and finite difference-based hybrid model to describe the evolution of nutrient concentration. The cells were modelled to proliferate based on nutrient uptake. Jiang et al. [15] developed a multi timescale-based model combining lattice Monte Carlo and CA-based methods for predicting the avascular tumour growth. The predictions from such models resemble closely the growth of multicellular spheroids without any drug testing parameters. Other models such as cancer stem cell driven cell growth models [17, 18] and tumour shrinkage model [19] have also been developed for simulating the tumour growth. These models have shown that the growth of multicellular spheroid can be captured using simpler reaction kinetics and force balances. However, numerical models, that are formulated based on experimental evidence or clinical data should be able to capture much more than just tumor growth. This aspect is currently inadequately addressed in existing literature.

In this study, we develop an experimental data driven numerical model capable of simulating drug response in spheroids. We interpret the drug chemotherapeutic mechanism to physically and stochastically definable parameters that can be directly implemented in the model. These parameters can be related to experimental observations and also can be quantified. The developed model enables us to analyse the spatially localized effects of the drug and the heterogeneity associated with a drug treated spheroid. By simulating multicellular spheroids, it should be possible to explore complex microenvironment settings, which would be otherwise not feasible to study using experiments. We consider the effects of two commercially available chemotherapy drugs, namely, *Taxol (paclitaxel)* and *cisplatin* on the spheroids of human cervical cancer HeLa cells. We performed experiments of growth dynamics and drug testing on the 3D HeLa spheroids. Using the experimental data, the model is tuned for the cell proliferation rate and changes in cellular oxidative stress levels and cell cycles arising from drug actions. The model simulations are validated against the spatial effects caused by the drugs used in the experiments. The model is further used for exploring temporal effects of combinatorial treatments on the simulated and experimental spheroids.

## Methods

### Cell culture

HeLa-C3 cells were generated by transfecting HeLa cells with sensor C3 plasmid encoding for a fluorescence resonance energy transfer (FRET)-based sensor for detecting caspase-3 activation [20]. HeLa-C3 cells were maintained in Dulbecco’s modified Eagle medium (DMEM, Thermo Fisher Scientific, USA) supplemented with 10% fetal bovine serum (FBS, Hyclone, USA) and 1% penicillin/streptomycin (PS, Thermo Fisher Scientific). The spheroid cultures were maintained in DMEM with 20% FBS and 1% PS.

HeLa-C3 cells were chosen as the model cell line due to the incorporated presence of fluorescence resonance energy transfer (FRET)-based biosensor. This enabled us to differentiate and estimate apoptosis and necrosis in the cells in real time. Additionally, their ability to readily form 3D spheroids and their susceptibility to both paclitaxel and cisplatin made them an excellent candidate for our study.

### 3D HeLa-C3 spheroid cell proliferation assay

The 3D culture was performed as previously described [7]. Non-adhesive round bottom 96-well plates (Sigma Aldrich, USA) were first pre-coated with 1% pluronic-F127 (Sigma Aldrich) before seeding HeLa-C3 cells. The plates were then centrifuged at 1000 g for 5 min in the Sorvall Legend XTR Centrifuge (Thermo Fisher Scientific) and left to incubate at 37^o^ C and 5% CO_2_. The medium was changed every day to ensure maximal oxygen and nutrient penetration into the spheroid. Prestoblue (Invitrogen) was added to the cells for each day and the absorbance was measured 2 h post addition of reagent. The radius of the spheroids was measured from 10 spheroid samples.

### Chemotherapeutic response with HeLa-C3 spheroids

Two hundred newton meter of paclitaxel and 10 μM of cisplatin were added to the spheroids 24 h after the seeding of 1500 HeLa-C3 cells. The concentration of Taxol and cisplatin exposed to the spheroids were assessed every 24 h. Spheroids were stained with propidium iodide (red) and images were captured with an inverted fluorescence microscope (Carl Zeiss, Thornwood, USA) attached to a SPOT CCD camera (Diagnostic Instruments, Sterling Heights, USA). FRET images were captured with dual filters of YFP and CFP under the same excitation (436 ± 10 nm) while PI images were captured with the RFP filter. Images collected from the filter sets were merged in Image Pro Plus (Media Cybernetics, Silver Spring, USA) to obtain FRET images of live (green), apoptotic (cyan) or dead (red) HeLa-C3 cells. Prestoblue was added to the cells and the absorbance at 570 nm and 600 nm were measured with the SpectraMax M5 Microplate Reader (Molecular Device) 2 h post addition of the reagent. For every individual well, the absorbance at 570 nm was subtracted by the absorbance at 600 nm to obtain sample readings before subtracting the average value of the control well.

### H&E staining

3D spheroids were fixed with formalin, processed and embedded in paraffin using standard protocols (Leica). Hematoxylin and eosin (H&E) stains were performed on 5-μm sections of the spheroids. Standard protocols were used, and spheroids were captured with a color camera (AxioCam 506, Carl Zeiss).

### Model description

#### Tumour cell proliferation and apoptosis

We use a 151x151x151 3D lattice simulation domain in which each lattice site can be occupied by fixed mass of cells. At the start of the simulation, t = 0, the domain is filled with a crucial growth nutrient, glutamine [21] of concentration, *C*_*g*_ and diffusion coefficient, *D*_*g*_. A cluster of cells is placed at the centre of the lattice domain. The cells consume glutamine at their lattice point at a rate r_c_, which is dependent on its mass *M*_*c*_, metabolic maintenance coefficient ‘*m*’, specific growth rate ‘ *K*_*cg*_ ’, half saturation coefficient ‘*S*’ and yield coefficient ‘*Y*’ as described in eq. 1. Time variation of glutamine concentration at different lattice points is modelled by the reaction-diffusion equation (eq. 2). In the corresponding experiments, we replace the spent glutamine each day with fresh glutamine from new medium. Therefore, glutamine is available in excess throughout the duration of the experiments. In a similar fashion, glutamine solute is modelled as a constant source boundary on all sides of the simulation domain. The open source finite volume method-based solver, FiPy [22] (3.1.1), is used to simulate the steady state reaction-diffusion of glutamine within the domain. The simulation time taken to reach steady state concentration is assumed to be 1 hour, after which cells proliferate, die or move. The cells uptake the surrounding glutamine and increase in mass after expending a part of the uptake proportional to the constant cell metabolic maintenance factor (*m*), governed by the Monod eqs. 1 and 3. The Monod kinetics used in the model ensures glutamine availability-dependant growth of the spheroids in the simulations. This closely mimics the nutrient limitation governed tumor growth observed in experiments. The daughter cell placement rules in the model may however give rise to growth patterns different from experimental observations. To overcome this limitation, multiple simulation runs with fixed parameters were carried out and the average growth observations are reported.

It is assumed that the cell mass at a lattice site divides when the mass doubles its initial value *(M*_*c*, *ini*_)*.* At the instance after division, the daughter cell is placed at a random location within the radius of 2.5 lattice sites from the mother cell. If no space is available within the 2.5 lattice radius, then the cell mass will not divide. If the concentration of glutamine decreases to a level below the prescribed threshold ‘ *C*_*gmin*_ ’, the cell can neither grow nor satisfy the metabolic maintenance cost, then the cell is assumed to undergo apoptotic stress damage ‘ *d*_*t*_ ’. The stress evolves with time (eq. 4 and 5) until it reaches the critical apoptotic stress value (*d*_*apoptosis*_). Cells having cumulative stress damage *d(t)* higher than *d*_*apoptosis*_ are declared as dead cells (eq. 6), which continue to occupy the same lattice site for 4 hours. After 4 hours (clearing time, *c*_*t*_) the dead cells are cleared from the system, by allowing the cells directly above the empty sites to move and occupy the space. This replicates the experimental and clinical observation of temporal shrinkage in tumour size.

1 $$ {\mathrm{r}}_{\mathrm{c}}\left({\mathrm{C}}_{\mathrm{g}},{\mathrm{M}}_{\mathrm{C}}\right)=\frac{\left(\frac{{\mathrm{K}}_{\mathrm{c}\mathrm{g}}}{\mathrm{Y}}+\mathrm{m}\right){\mathrm{M}}_{\mathrm{c}}{\mathrm{C}}_{\mathrm{g}}}{\mathrm{S}+{\mathrm{C}}_{\mathrm{g}}} $$

2 $$ \frac{\mathit{\partial}{C}_g}{\mathit{\partial t}}={D}_g\left(\frac{{\mathit{\partial}}^2{C}_g}{\mathit{\partial}{x}^2}+\frac{{\mathit{\partial}}^2{C}_g}{\mathit{\partial}{y}^2}+\frac{{\mathit{\partial}}^2{C}_g}{\mathit{\partial}{z}^2}\right)-{r}_c $$

3 $$ \frac{d{M}_c}{dt}={M}_c{K}_{cg}\frac{C_g}{S+{C}_g} $$

4 $$ \mathrm{d}\left(\mathrm{t}\right)={\sum}_{\mathrm{t}}{\mathrm{d}}_{\mathrm{t}} $$

5 $$ {\mathrm{d}}_{\mathrm{t}}=\left\{\begin{array}{c}1,\mathrm{when}\ {\mathrm{C}}_{\mathrm{g}}<{\mathrm{C}}_{\mathrm{g}\mathrm{min}}\\ {}0,\mathrm{when}\ {\mathrm{C}}_{\mathrm{g}}\ge {\mathrm{C}}_{\mathrm{g}\mathrm{min}}\end{array}\right\} $$

6 $$ {\mathrm{M}}_{\mathrm{c}}=0,\mathrm{when}\ \mathrm{d}\left(\mathrm{t}\right)>{\mathrm{d}}_{\mathrm{apoptosis}} $$

#### Effects of cisplatin on cells

Cisplatin is primarily considered to mediate cell death by targeting fast proliferating cells. It damages cellular DNA by cross linking the DNA of the cells to form DNA-cisplatin adducts [23, 24]. The nucleotide excision repair (NER) pathway is activated in order to remove these adducts; however, upon failure of repair, cell apoptosis is triggered in mitotic cells and the dividing cells undergo cell death. Yet, recent studies have indicated that the cytotoxicity of cisplatin is not only limited to replicating cells. Cisplatin may elicit cell death independently of DNA damage via oxidative stress [25, 26] at three primary locations [27]. The first is the plasma membrane, where NOX induces reactive oxygen species (ROS) [28] which in turn trigger Fas aggregation [29] and influence the activity of membrane channels such as Ca^2+^ channels [28, 30]. Engagement and clustering of Fas receptor mediates caspase activation, while Ca^2+^ influx changes electrolyte capacities of the cells [28], which ultimately leads to cell apoptosis. Second, cytoplasm produces cellular superoxide formation through the interaction of cisplatin with nuclear DNA, thereby activating an important regulator of cell proliferation, differentiation and survival, the MAPK pathway [27, 31]. The final and most important location is the mitochondrion, which generates free radical during oxidative phosphorylation and is one of the most important source of endogenous ROS [27]. Treatment of cisplatin in NER deficient cells displayed similar mitochondrial ROS generation as NER proficient cells, demonstrating cisplatin-induced mitochondrial ROS production regardless of the ability of cells to repair cisplatin-induced nuclear DNA damage [25]. At high doses of 10 μM or more, loss of mitochondrial membrane permeabilization, activation of BAK and caspases [32] are triggered by superoxide formation induced by cisplatin [25, 29], which is effectively blocked by antioxidants Manganese superoxide dismutase (MnSOD) and glutathione [29, 33] and reactive oxygen species (ROS) scavengers [32].

7 $$ \frac{\partial {\mathrm{C}}_{\mathrm{cis}}}{\mathrm{\partial t}}={\mathrm{D}}_{\mathrm{cis}}\left(\frac{\partial^2{\mathrm{C}}_{\mathrm{cis}}}{\partial {\mathrm{x}}^2}+\frac{\partial^2{\mathrm{C}}_{\mathrm{cis}}}{\partial {\mathrm{y}}^2}+\frac{\partial^2{\mathrm{C}}_{\mathrm{cis}}}{\partial {\mathrm{z}}^2}\right)-{\mathrm{K}}_{\mathrm{intra}}{\updelta}_{\mathrm{cell}}\ \left({\mathrm{C}}_{\mathrm{cis}}-{\mathrm{C}}_{\mathrm{intra}\mathrm{cis}}\right) $$

8 $$ \frac{\partial {\mathrm{C}}_{\mathrm{intra}\mathrm{cis}}}{\mathrm{\partial t}}={\mathrm{K}}_{\mathrm{intra}}{\updelta}_{\mathrm{cell}}\ \left({\mathrm{C}}_{\mathrm{cis}}-{\mathrm{C}}_{\mathrm{intra}\mathrm{cis}}\right)-{\mathrm{K}}_{\mathrm{bonding}}{\updelta}_{\mathrm{cell}}{\mathrm{C}}_{\mathrm{intra}\mathrm{cis}}-{\mathrm{K}}_{\mathrm{deg}}{\updelta}_{\mathrm{cell}}{\mathrm{C}}_{\mathrm{intra}\mathrm{cis}} $$

9 $$ \frac{\partial {\mathrm{C}}_{\mathrm{adduct}}}{\mathrm{\partial t}}={\mathrm{K}}_{\mathrm{bonding}}{\mathrm{C}}_{\mathrm{intracis}} $$

10 $$ {\updelta}_{\mathrm{c}\mathrm{ell}}=\left\{\begin{array}{c}0,\mathrm{where}\ {\mathrm{M}}_{\mathrm{c}}=0\\ {}1,\mathrm{where}\ {\mathrm{M}}_{\mathrm{c}}>0\end{array}\right\}\# $$

11 $$ {\mathrm{C}}_{\mathrm{adduct}}=\left\{\begin{array}{c}0,\mathrm{if}\ {\mathrm{P}}_{\mathrm{rep}}\left(\mathrm{t}\ \right)<\mathrm{R}\\ {}{\mathrm{C}}_{\mathrm{adduct}},\mathrm{if}\ {\mathrm{P}}_{\mathrm{rep}}\left(\mathrm{t}\right)\ge \mathrm{R}\end{array}\right\} $$

12 $$ {\displaystyle \begin{array}{c}{\mathrm{C}}_{\mathrm{l}}={\mathrm{C}}_{\mathrm{cismax}}\left(\frac{1}{\mathrm{A}+{\mathrm{Be}}^{-{\mathrm{t}}_{\mathrm{g}}}}\right)\\ {}\ \mathrm{here},{\mathrm{t}}_{\mathrm{g}}=\left({\mathrm{t}}_{\mathrm{m}}-{\mathrm{t}}_{\mathrm{cg}}\right)\ \end{array}} $$

13 $$ {\mathrm{M}}_{\mathrm{c}}=0,\mathrm{when}\ {\mathrm{C}}_{\mathrm{adduct}}>{\mathrm{C}}_{\mathrm{l}} $$

The effects of cisplatin on the cells are numerically modelled by assuming that cancer cells experiencing oxidative stress suffer DNA damage upon treatment with cisplatin. DNA damage builds-up during the course of the treatment. Therefore, ROS formation in the model correlates with the quantity of cisplatin uptake by the cells. The drug uptake is modelled by eqs. 7–10, where *A* and *B* are constants. To model the uptake of cisplatin more accurately, cisplatin concentration is split into two parts, cisplatin concentration outside the cell or extracellular concentration *(C*_*cis*_), with diffusion coefficient (*D*_*cis*_) and concentration inside the cell or intracellular concentration (*C*_*intracis*_) with passive diffusion rate constant (*K*_*intra*_). The intracellular cisplatin degrades based on the degradation rate constant (*K*_*deg*_). The rate of formation of DNA adducts (*C*_*adduct*_) is assumed to be directly proportional to the intracellular concentration with a rate constant (*K*_*bonding*_). The cells attempt to remove excess ROS via their endogenous antioxidant defence mechanism in-vivo and in-vitro. In accordance with these experimental observations, in the model, the cells eliminate the adducts formed based on the probability ‘*R*’ and *P*_*rep*_(*t*) as shown in eq. 11. The cells in the model are assumed to repair themselves with equal probability ‘*R’*. Cells exposed to cisplatin respond differently based on their exposure times to the drug. Therefore, an apparent lethal concentration *C*_*l*_ is calculated based on eq. 12, which is dependent on the cell’s cumulative generational age *t*_*cg*_, maximum generational age for mutation *t*_*m*_, and the age-independent maximum adduct concentration *C*_*cismax*_ as shown in eq. 12. Cumulative generational age is the total existing time of the cell’s generation. The cumulative age is divided equally between the daughter cells at the time of division. In the model, we assume no mutation of the cells and therefore do not consider the effects of acquired drug resistance to cell lysis. Therefore, we include the maximum generational age parameter *t*_*m*_, which imposes maximum probability of cell lysis for a long-lasting cell generation capable of mutating. This sets an artificial time boundary after which none of cells are allowed to exist in the simulation and accumulate mutations. If the adduct concentration *C*_*adduct*_ is larger than the permissible lethal adduct concentration *C*_*l*_, after all possible cell repair processes, the cells undergoes apoptosis. However, if the cell is able to overcome cisplatin cytotoxicity, in-vivo or in-vitro, with its NER pathway, and reduce ROS through its antioxidant defence, the cell mass divides with the oxidative stress level lesser than the lethal threshold. This phenomenon is modelled in such a way that both the daughter cell and the parent cell do not inherit any DNA damage, that is, *C*_*adduct*_ and *C*_*cis*_ are reset.

#### Effects of Taxol on cells

Cells uptake Taxol from their surroundings through passive diffusion [34]. Taxol uptake is modelled by eqs. (14–16). Taxol is separated into three different components based on its localization [35], concentration outside the cell *C*_*tax*_, concentration inside the cell *C*_*intratax*_ and concentration of Taxol bound to intra-cellular components *C*_*bound*_. A part of extracellular Taxol binds to the proteins in the medium, which is quantified by the third term in eq. 14, where, *k*_*b*_ is the rate constant for protein binding, *B*_*m*_ and *K*_*p*_ are the Michaelis-Menten constants for protein binding. The drug molecules remaining after protein binding move from the medium into the cells through passive diffusion with clearance *c*_*l*_. Similar to the extracellular protein binding, the intracellular Taxol binds to saturable and non-saturable sites of the cellular components as quantified by the second and third terms in eq. 15 respectively, where *B*_*mc*_ and *K*_*pc*_ are the Michaelis-Menten constants for cellular component binding and *N*_*s*_ is the rate constant for binding to non-saturable sites. The bound Taxol (*C*_*bound*_) then acts on the cells in their G2/M phase by stabilising the microtubules. This causes mitotic arrest of the cells at the spindle checkpoint [36], resulting in cell apoptosis. There are reports [37, 38] on the formation of multipolar spindles in the cells post Taxol treatment. It is important to note that Taxol is found to affect only the cells that have undergone mitosis in the presence of drug and the cells that are in their interphase during the drug treatment. Similar to the progressive, concentration based, lethal effects of cisplatin on the cells, the activity of Taxol is dependent on the intracellular concentration levels of the drug [39].

To mimic these mechanisms of actions, in our simulation model, Taxol is assumed to affect only the parent cells that have divided in the presence of the drug. This assumption is applicable through the generation of the cells, meaning, the daughter cells from a cell division will be affected by the drug only if they have been exhibited their G2/M phase in the presence of the drug. This assumption makes sure that the effects of the drug do not carry over to the future generations after cell division as observed by Gascoigne [40]. Cells in the simulation whose mass is 1.5 times higher than the initial cell mass (*M*_*c*, *ini*_) are tagged as cells in their G2/M phase (*G*_*2cell*_). Cell apoptosis is modelled in a stochastic fashion in accordance with experimental observations [40]. Cells in the simulations are chosen for apoptosis only if their bound Taxol concentration is above lethal Taxol concentration (*C*_*lethal*_*)* and the chosen cells are declared dead if the probability of cell survival (*P*_*sur*_(*t*)) is lower than the fixed cell apoptosis probability (*R*_*d*_).

14 $$ \frac{\partial {\mathrm{C}}_{\mathrm{tax}}}{\mathrm{\partial t}}={\mathrm{D}}_{\mathrm{tax}}\left(\frac{\partial^2{\mathrm{C}}_{\mathrm{tax}}}{\partial {\mathrm{x}}^2}+\frac{\partial^2{\mathrm{C}}_{\mathrm{tax}}}{\partial {\mathrm{y}}^2}+\frac{\partial^2{\mathrm{C}}_{\mathrm{tax}}}{\partial {\mathrm{z}}^2}\right)-{\mathrm{c}}_{\mathrm{l}}{\updelta}_{\mathrm{c}\mathrm{ell}}\left({\mathrm{C}}_{\mathrm{tax}}-{\mathrm{C}}_{\mathrm{intratax}}\right)-{\mathrm{k}}_{\mathrm{b}}\frac{{\mathrm{B}}_{\mathrm{m}}}{{\mathrm{K}}_{\mathrm{p}}+{\mathrm{C}}_{\mathrm{tax}}}{\mathrm{C}}_{\mathrm{tax}} $$

15 $$ \frac{\mathit{\partial}{C}_{intratax}}{\mathit{\partial t}}={c}_l{\delta}_{cell}\left({C}_{tax}-{C}_{intratax}\right)-{k}_b\frac{B_{mc}}{K_{pc}+{C}_{intratax}}{C}_{intratax}-{N}_s{C}_{intratax} $$

16 $$ \frac{\partial {\mathrm{C}}_{\mathrm{b}\mathrm{ound}}}{\mathrm{\partial t}}={\mathrm{k}}_{\mathrm{b}}\frac{{\mathrm{B}}_{\mathrm{mc}}}{{\mathrm{K}}_{\mathrm{pc}}+{\mathrm{C}}_{\mathrm{intratax}}}{\mathrm{C}}_{\mathrm{intratax}}+{\mathrm{N}}_{\mathrm{s}}{\mathrm{C}}_{\mathrm{intratax}} $$

17 $$ {\mathrm{G}}_{2\mathrm{cell}}=\left\{\begin{array}{c}0,\mathrm{where}\ {\mathrm{M}}_{\mathrm{c}}<1.{5\mathrm{M}}_{\mathrm{c},\mathrm{ini}}\ \\ {}1,\mathrm{where}\ {\mathrm{M}}_{\mathrm{c}}\ge 1.{5\mathrm{M}}_{\mathrm{c},\mathrm{ini}}\ \end{array}\right\} $$

18 $$ {\mathrm{M}}_{\mathrm{c}}=\left\{\begin{array}{c}0,\mathrm{if}\ {\mathrm{C}}_{\mathrm{bound}}\ge {\mathrm{C}}_{\mathrm{lethal}},{\mathrm{P}}_{\mathrm{sur}}\left(\mathrm{t}\right)<{\mathrm{R}}_{\mathrm{d}}\ \mathrm{and}\ {\mathrm{G}}_{2\mathrm{cell}}=1\ \\ {}{\mathrm{M}}_{\mathrm{c}},\mathrm{if}\ {\mathrm{C}}_{\mathrm{bound}}<{\mathrm{C}}_{\mathrm{lethal}}\end{array}\right\} $$

## Results

### HeLa-C3 cell proliferation profiles

**Fig. 1:**
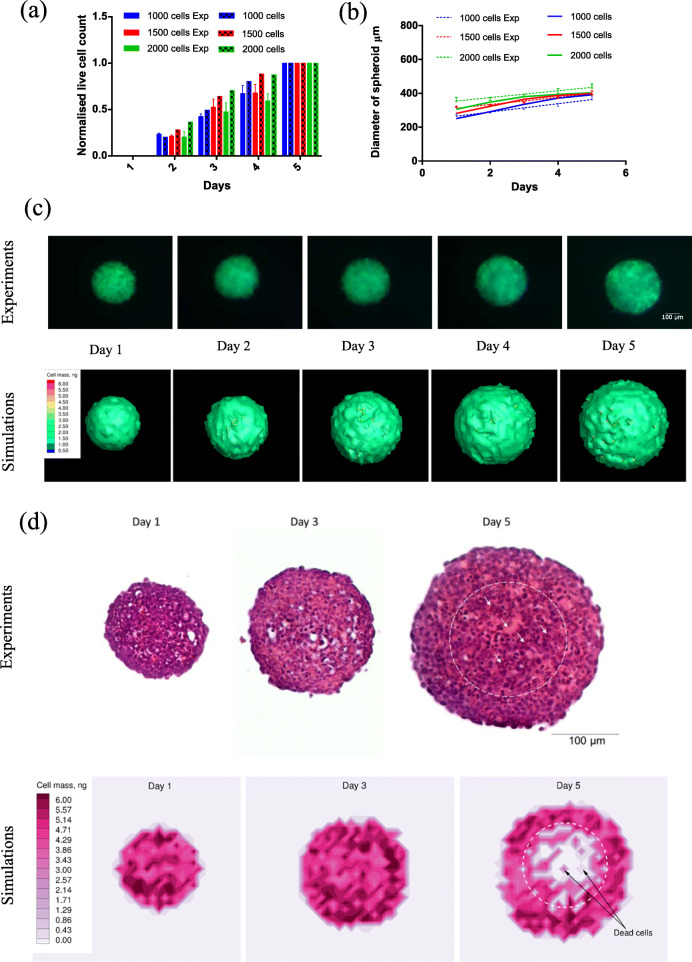
Cell proliferation in absence of drug treatment. Comparison of experimental data and simulation results for (**a**) normalized cell viability of HeLa-C3 cells, (**b**) spheroid diameter, (**c**) shape evolution of spheroids and (**d**) sectioned spheroids at different time points. Green spots in the experimental panel of (**c**) indicate live cells and cyan spots indicate dead cells. GraphPad Prism software v6.01 was used to generate plots (**a**) and (**b**). Tecplot 360 2018 R1 was used to generate plots (**c**) and (**d**) simulations subplots

Experimental quantification and validation of simulation profiles for proliferation of HeLa-C3 cells and increases in spheroid diameter are prerequisites for subsequent drug efficacy estimations. Hence, experiments were carried out using three different initial cell seedings, 1000, 1500 and 2000 cells and their proliferation rates are summarized in Fig. 1a. For cell seedings with 1000 and 1500 cells, the experimental results and simulated values are numerically similar to each other at different time points within acceptable error margins (approximately ±5%). However, for initial cell seeding of 2000 cells, the mismatch of cell viability between the experimental data and simulation results is high. This is due to the fixed domain size introduced in the simulations, which artificially constrains the available free glutamine in the simulated medium. In case of experiments, glutamine is an excess solute. Due to the limited availability of glutamine for a large simulated tumor, the cell growth saturates. This numerical artefact is well reflected in the simulated diameters of the spheroids (Fig. 1b), where the diameter increase is linear at the start of simulation and saturates as the simulation progresses. Therefore, to avoid any artificial growth saturation effects, simulations and experiments with 1500 cell seedings are used in all drug and parameter estimation studies. The values of model simulation parameters used in the study are listed in Table 1.

**Table 1:** Summary of model simulation parameters

Parameter	Notation	Value	Units
Concentration of glutamine (maximum)	퐶_푔_	2.0	mM
Diffusivity of glutamine	퐷_푔_	7.6 × 10^− 10^	m^2^ s^− 1^
Initial mass of cell	푀_푐,푖푛푖_	3.3 × 10^− 12^	kg
Metabolic maintenance coefficient	푚	2.0 × 10^− 8^	s^− 1^
Max Specific growth rate	퐾_푐푔_	8.75 × 10^− 6^	s^− 1^
Half saturation coefficient	푆	0.1	mol m^− 3^
Yield coefficient	푌	100	
Critical apoptotic stress value	푑_푎푝표푝푡표푠푖푠_	8–14	h
Threshold concentration of glutamine	퐶_푔푚푖푛_	0.6	mM
Cell clearing time	푐_푡_	4	h
Maximum concentration of cisplatin	퐶_푐푖푠_	10.0	μM
Diffusivity of cisplatin	퐷_푐푖푠_	8.2 × 10^−10^	m^2^ s^−1^
Diffusivity of cisplatin (Intra-spheroidal)	퐷_푐푖푠_	1.78 × 10^− 11^	m^2^ s^− 1^
Cisplatin degradation rate constant	퐾_푑푒푔_	1 × 10^− 4^	s^− 1^
Cisplatin bonding rate constant	퐾_푏표푛푑푖푛푔_	3 × 10^− 3^	s^− 1^
Maximum generational age	푡_푚_	8	days
Maximum age-independent adduct concentration	퐶_푐푖푠푚푎푥_	2.8 × 10^− 3^	μM
Passive diffusion rate constant of cisplatin	퐾_푖푛푡푟푎_	20.96	h^−1^
Concentration of Taxol (maximum)	퐶_푡푎푥_	200.0	nM
Diffusivity of Taxol (Intra-spheroidal)	퐷_푡푎푥_	9.25 × 10^−12^	m^2^ s^− 1^
Diffusivity of Taxol	퐷_푡푎푥_	4.26 × 10–^10^	m^2^ s^− 1^
Taxol drug clearance	푐_푙_	1.91 × 10^− 1^	s^− 1^
Lethal Taxol concentration	퐶_푙푒푡ℎ푎푙_	1.5 × 10^− 3^	nM
Drug binding rate constant of Taxol	푘_푏_	3.0 × 10^− 6^	s^− 1^
Drug binding rate constant of Taxol to unsaturable sites	푁_푠_	4.1 × 10^− 5^	s^− 1^
Michaelis-Menten constant for extracellular protein binding	퐵_푚_	3.94 × 10^− 3^	mol m^− 3^
Michaelis-Menten constant for cellular component binding	퐵_푚푐_	5.92 × 10^− 2^	mol m^− 3^
Half saturation coefficient for extracellular protein binding	퐾_푝_	7.81 × 10^− 4^	mol m^− 3^
Half saturation coefficient for cellular component binding	퐾_푝푐_	4.93 × 10^− 6^	mol m^− 3^

The shape variations in tumor growth is summarized in Fig. 1c. Spheroids retain their spherical shapes with little localized distortions throughout the observation duration in both simulation and experiment. The green spots in the experiments panel, Fig. 1c, indicate live cells and cyan spots indicate cells undergoing apoptosis. The cyan spots are seen to increase at the core of the spheroids as time progresses suggesting the formation of necrotic core. The simulated spheroids in Fig. 1c also exhibit a similar color profile to that of the experiments. However, because of variations in hue and translucency of simulated and experimental spheroids, sectioning of spheroids is necessary to properly visualize the formation of necrotic core in the spheroids. Sectioned and stained spheroids are shown in Fig. 1d experiments panel. There are empty spots (white color) present in the sectioned spheroids in both the experiments and simulations. These spots indicate the absence of live cells at those positions, indicating improper packing of cells within the tumor. This improper packing can be a result of slow cell migration/motility to occupy a lysed cell’s spot in the experiments and an outcome of the discrete clearing time *c*_*t*_ used in the simulations. In the sectioned simulation contours, the cells undergoing apoptosis, because of value interpolation, will have a value ranging from 3 ng to 0 ng color bars. The necrotic cells in the experimental images are indicated by white arrows. The dotted circle in the experimental and simulated sectioned images indicates the extent of necrotic zone in the spheroids. Necrotic core forms as a result of diffusion limitation of glutamine resulting from increased spheroid sizes at later stages of growth.

### Spatial effects of cisplatin on HeLa-C3 spheroids

**Fig. 2:**
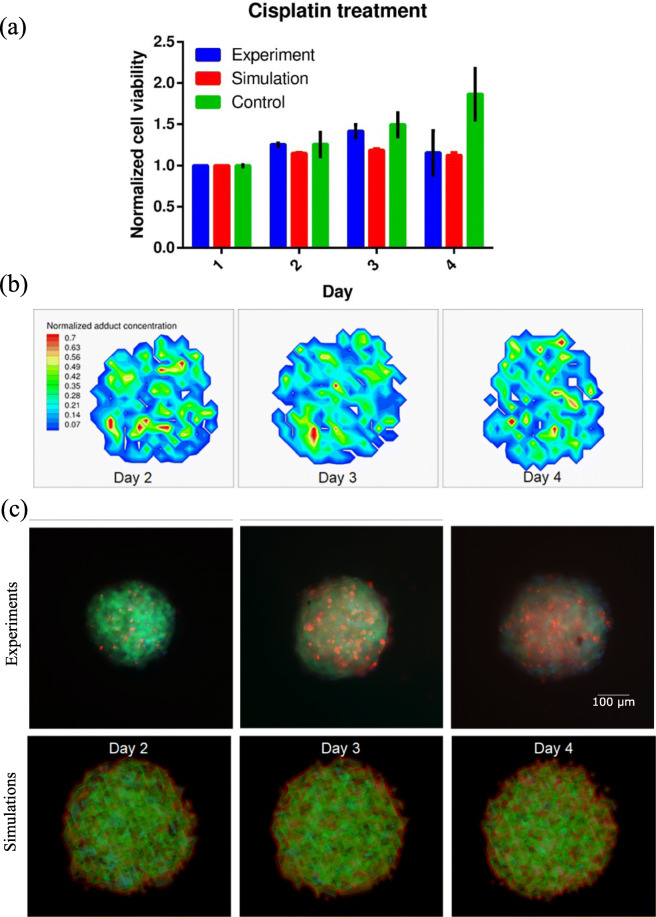
Cell proliferation under cisplatin treatment. **a** Comparison of experimental data and simulation results for normalized cell viability of HeLa-C3 cells at different time points, **b** Contours plots of adduct concentration in the cells at the centre slice of simulated spheroid at different time points and (**c**) Comparison of experimental data and simulation results for spatial activity of cisplatin at different time points. Green spots in the experimental panel of (**c**) indicate live cells, cyan spots indicate dead cells due to apoptosis alone and red spots indicate dead cells from apoptosis triggered by cisplatin. Green spots in the simulation panel of (**c**) indicate live cells and red spots indicate dead cells. GraphPad Prism v6.01 was used to generate plot (**a**). Tecplot 360 2018 R1 was used to generate plots (**b**) and (**c**) simulations subplot

An initial cell seeding of 1500 cells was selected as the starting configuration for all simulations incorporating drug treatment. To avoid any undesirable artifacts arising from drug depletion due to cell uptake, cisplatin used in the experiments was replaced at the end of every day. The differential response of cells to various cisplatin concentrations is simulated using the numerical model for cisplatin described in the model description. To avoid overfitting of the numerical model, most of the parameters used in the cisplatin simulations were adapted from experimental values in the literature. Only two values, lethal adduct concentration *C*_*l*_ and cell repair probability *R*, were free (adjustable) parameters in the simulation. These values were tuned through trial and error process with multiple simulation runs, to obtain the optimum parameter values that represent the experimental results. The comparison of experimental and simulated cell viability values is summarized in Fig. 2a. Cell viability values for cisplatin treated spheroids was always lower than the control values in both experiments and simulations. The simulated values of normalized cell viability are found to correspond to the experimental values at all time points as shown in Fig. 2a. To further understand the spatial variations of cell viability, the concentration contours of normalized adduct concentration are plotted at different time points as shown in Fig. 2b. The spatial adduct concentration is found to be randomly distributed throughout the spheroid at all time points. Peak adduct concentrations (red color) are observed at random locations within the spheroids and do not exhibit any localization effects.

Three different fluorescence indicators were used in experiments to differentiate the effect of cisplatin and cell apoptosis in spheroids. The green spots in the experiments panel of Fig. 2c indicate the live cells, cyan spots indicate apoptotic cells and red spots indicate cells affected by cisplatin. In Fig. 2c, from the experimental panel it is evident that drug activity increases with time progress as indicated by increase in red spots. Results summarized in the simulation panel are found to exhibit multiple dead cells (red color) at random locations within the spheroid. This observation is further confirmed by the microscopy images with color-coded cells in the spheroids as shown in the experiments panel of Fig. 2c. Red dots (cells affected by cisplatin) are spotted all over the volume of spheroids indicating the spatially randomized effect of cisplatin. From these observations it is clear that the effects of cisplatin are not localized to specific cell populations in the spheroid.

It is also known that cisplatin’s toxicity is more effective at higher concentrations, this means that cells exposed to higher cisplatin concentration should be affected earlier than those residing in low concentrations. From the simulations it is observed that the drug penetrates evenly throughout the volume of the spheroid. This should mean that intracellular drug concentration and consequently the adduct concentration must be almost uniform throughout the spheroid, but the simulations do not conform to this logic, adduct concentration is not uniformly distributed within the spheroid. As seen in Fig. 2b the distribution of adduct concentration within the spheroids is randomly distributed. Based on eqs. 7,8 and 9, the adduct concentration (*C*_*adduct*_) is dependent on the diffusivity of cisplatin, passive diffusion rate constant (*K*_*intra*_) and degradation rate (*K*_*deg*_). All these values are constants and should therefore result in uniform concentration profile development in the domain. A stochastic parameter that can drastically alter the distribution profile is the cell repair probability *(R*) listed in eq. 11, which is an inherent property of the cell. This property enables efflux of any adduct formed post cell repair, thereby altering the distribution dynamics throughout the spheroid. Any spatially localized segregation of this property will result in preferential action or inaction of cisplatin on this local population. In fact, cell repair has been largely implicated in cisplatin drug resistance development [41–44]. In contrast to drug resistance development, cell repair property has been exploited to increase efficacy of cisplatin treatment in recent studies [42, 45, 46] . Cell repair property has also been implicated in pathological complete response of triple-negative breast cancers [47]. In cell populations without any abnormal cell repair characteristics, cisplatin will not exhibit any structurally preferential effects over the spheroid volume.

### Spatial effects of Taxol on HeLa-C3 spheroids

**Fig. 3:**
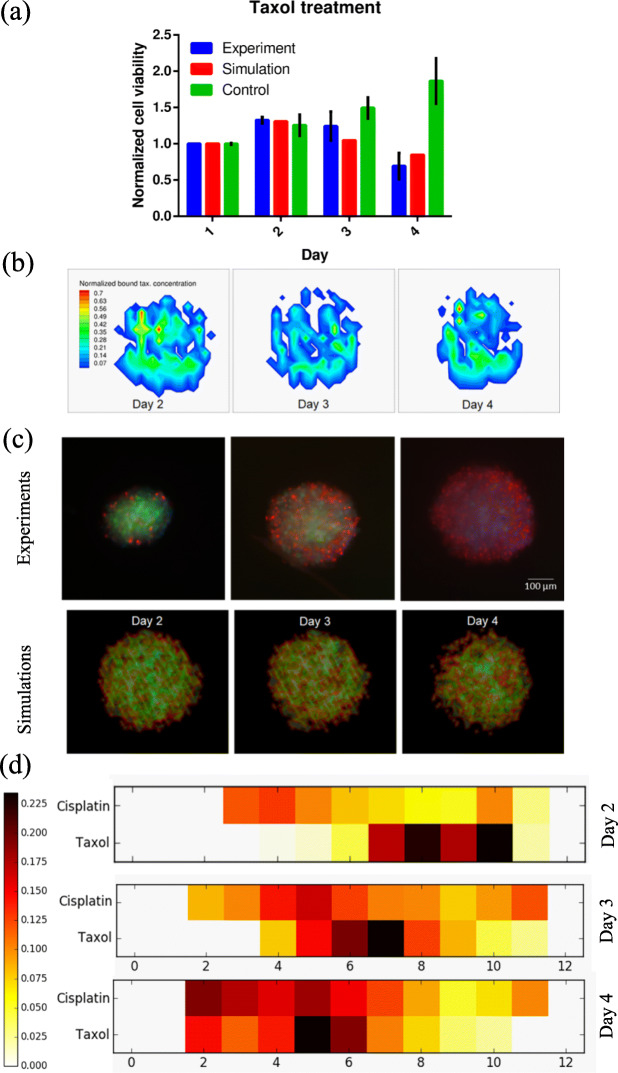
Cell proliferation under Taxol (paclitaxel) drug treatment. **a** Comparison of experimental data and simulation results for normalized cell viability of HeLa-C3 cells at different time points, (**b**) Contours plots of bound Taxol concentration in the cells at the centre slice of simulated spheroid at different time points, (**c**) Comparison of experimental data and simulation results for spatial activity of cisplatin at different time points and (**d**) Heat map of ratio of dead to live cells found at different areas of spheroids treated with cisplatin and Taxol at different time points. Green spots in the experimental panel of (**c**) indicate live cells, cyan spots indicate dead cells due to apoptosis alone and red spots indicate dead cells from apoptosis triggered by cisplatin. Green spots in the simulation panel of (**c**) indicate live cells and red spots indicate dead cells. The color bar in (**d**) indicates the dead to live cell ratio. GraphPad Prism v6.01 was used to generate plot (**a**). Tecplot 360 2018 R1 01 was used to generate plots (**b**) and (**c**) simulations subplot. Matplotlib v3.3.2 was used to generate plot (**d**)

The same cell seeding of 1500 cells used in cisplatin simulations and experiments was selected as the starting configuration for taxol drug treatment. Taxol used in the experiments was replaced at the end of every day to avoid any artificial artifacts. Similarly, the boundaries of the domain are fixed with constant concentration boundary conditions in the simulations. Cell cycle is indirectly modelled in Taxol simulations by considering the cell’s mass. Any growing cell whose mass is higher than 1.5 times its initial mass is considered to be in G2/M phase. Cell cycle phase is considered in the numerical model due to the mechanistic requirement of Taxol’s activity as explained in the model description. All parameters used in the Taxol model simulations were adapted from experimental values in the literature except for two values lethal Taxol concentration *C*_*lethal*_ and the fixed cell apoptosis probability (*R*_*d*_). Cell apoptosis probability is the same for all cells in the simulations. Therefore, all the cells exhibit same levels of resistance to Taxol activity. The two values were tuned through trial and error process with multiple simulation runs, to obtain the optimum parameter values that represent the experimental results. The comparison of experimental and simulated cell viability values is shown in Fig. 3a. Cell viability values for the Taxol treated spheroids in experiments and simulations are found to be within their each other’s variations and lower than the control values at all time points. Between cisplatin (Fig. 2a) and Taxol (Fig. 3a), Taxol is more effective with much lower cell viability values than cisplatin. To examine the spatial variations of cell viability, the concentration contours of normalized bound Taxol concentration are plotted at different time points as shown in Fig. 3b. From the contour plots it can be observed that as time progresses the fraction of cells with drug bound to cellular components decreases. This is because of the fast drug-induced apoptosis permeated by Taxol, as evident from the viability plots. Compared to cisplatin’s adduct concentration (Fig. 2b), Taxol’s cell bound concentration appears to be more homogenously distributed, barring a couple of hotspots at each time point. If Taxol’s efficacy is solely dependent on its cell bound concentration, then it should induce apoptosis in all cells in the spheroid irrespective of their positions. However, the experiments and simulations panel of Fig. 3c show that most of the cells along the periphery of the spheroid are affected. The experiments panel visually distinguishes the localization effect of Taxol and nutrient deficient cell apoptosis. Specifically, on day 4 a cyan core can be observed at the centre of the spheroid surrounded by concentric layers of red cells affected by Taxol. Similar to experimental observations, simulation images also show preferential action of Taxol along the periphery of the spheroids. To understand the reason behind such spatially preferential action of Taxol, the mechanism of Taxol action should be carefully considered. As discussed in the model description, Taxol only affects the cells that have previously undergone mitosis and are in G2/M phase of cell cycle. Cells that are present along exterior layers of the spheroids have a high likelihood of undergoing multiple cell divisions due to ready availability of glutamine in the surrounding. This increased frequency of cell mitosis at the periphery will result in high probability of cell apoptotic induction by Taxol. Thus, the efficacy of Taxol is affected by the nutrient concentration available to local population. The response of cells to Taxol is, therefore, not only dependent on the quantity of drugs bound to the cells, but also on their mitotic history and current stage of cell cycle.

To compare and contrast the spatial effects of cisplatin and Taxol on the cells in the spheroid, the simulated dead to live cell ratio should be quantified. The spatial domain is binned into twelve areas. These areas occupy the space between concentric circles with the increase in radius by one grid. Ratio of dead cells to live cells present within these binned areas is plotted as a heatmap at different timepoints as shown in Fig. 3d. For uniform representation, empty areas, (where, dead/live = 0/0) a value of 0.0 is used. At all timepoints, cisplatin is found to exhibit a homogenous dead to live cell distribution compared to Taxol. In both cisplatin and Taxol, the core of the spheroid (bin 1) is found to have a value of 0.0 indicating absence of dead cells, suggesting absence of necrotic core until day 4 because of decreased cell viability in the drug treated spheroids compared to control. From experimental data and simulation results we know that Taxol preferentially affects the cells in the periphery. This is once again confirmed by the appearance of dark patches at day 2 along zones 8,9 and 10 in Fig. 3d for Taxol. In addition to the local segregation of Taxol effect, a remarkable pattern occurs in Taxol treated spheroids with progress of time. This pattern is the inward shifting of high efficacy zones (dark color) as the simulation progresses. Taxol shows maximum efficacy in zones 8–10 on day 2, in zones 6–8 on day 3 and in zones 5–6 on day 4. These results show that Taxol affects the spheroid in a concentric manner with progress of time. Such a pattern emerges from the interplay of newly exposed layer of cells to the incoming nutrients and the binding action of Taxol. Excess glutamine promotes cell proliferation, while bound Taxol increases the probability of apoptosis induction in such frequently dividing cells.

### Temporal effects of drug scheduling on HeLa-C3 spheroids

**Fig. 4:**
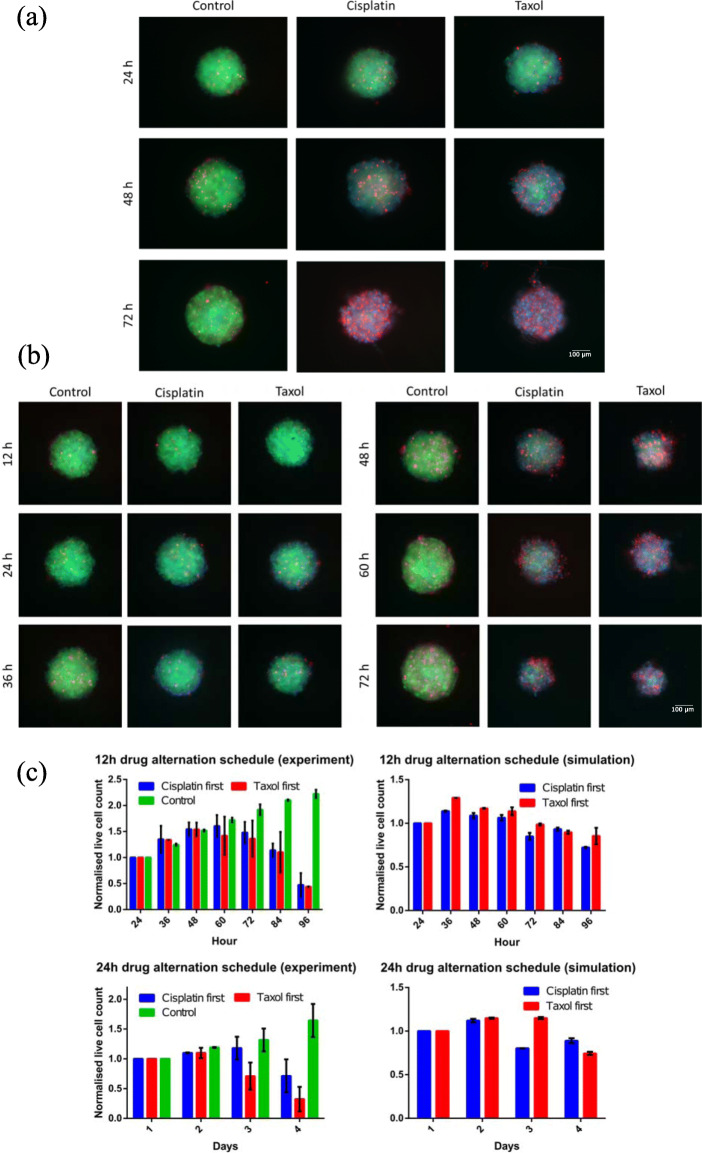
Summary of cell proliferation behaviours for different drug scheduling strategies. Experimental data/microscopy images of spheroids subjected to different treatment combinations at different time points for (**a**) 24-h alternate scheduling and (**b**) 12-h alternate scheduling, (**c**) Comparison of experimental data and simulation results for normalized cell viability of HeLa-C3 cells at different time points for 12-h and 24-h alternate treatment. The labels cisplatin and taxol in (**a**) and (**b**) indicate the initialisation drug or the drug used at the start of the scheduling after which the other drug was alternated, and the cycle was repeated at different time points. GraphPad Prism v6.01 was used to generate plot (**c**)

The unique spatial effects of cisplatin and Taxol assert that each drug has its own preferential sites of action where its efficacy is high. If these two drugs are used in a combinatorial manner and if the overlap of their high efficacy zones are minimized, then the outcomes from such combinations should surmount the efficacy of individual drug treatments. In fact, such combinatorial treatments are quite common for treating carcinoma patients. However, most of their combinatorial effects have been attributed to synergistic effects arising from the action of drugs in tandem. In addition to these synergistic effects, spatial effects must play a crucial part in increased efficacy of combinatorial treatments. For instance, consider an alternating Taxol-cisplatin treatment schedule. In this treatment schedule, initially the cells are treated with Taxol for a fixed duration followed by cisplatin treatment for the same fixed duration and the cycle is repeated. According to our findings, Taxol should first affect the outer layers of the tumor. Then, a lag sets in for inward Taxol effect propagation, since the cells in the layer which is in immediate proximity should take their time for proliferation. Introduction of cisplatin during this lag interval will help eliminate other cells in the tumor and also give the required time for cell proliferation. Subsequent Taxol treatment should result in immediate elimination of the penultimate layer, thereby making the combinatorial process highly efficient. Therefore, to analyse these temporal effects, in addition to the studies on spatial effects of the drug on HeLa-C3 spheroids, the temporal effect of combinatorial drug treatment was studied experimentally and numerically. The results from these studies are summarized in Fig. 4. Two different schemes of drug scheduling were implemented, alternating treatment of cisplatin and Taxol for (i) 24 h and (ii) 12 h. The schemes are further split into two strategies, (a) treatment initialized with Taxol and (b) treatment initialized with cisplatin. In Fig. 4a and b the tags, cisplatin/Taxol indicate the treatment strategy and the time points indicate the scheduling. For example, a 24-h scheme initialized with Taxol will be as follows; day 1 – Taxol treatment, day 2 – cisplatin treatment (with Taxol been removed) and day 3 – Taxol treatment (with cisplatin been removed). The removal of the treated drug at the end of a treatment cycle ensures that treated drug effects are not carried over to the next cycle.

For the spheroid treatment initialized with Taxol, the cell apoptosis effects on day 2 are predominantly found along the periphery compared to the volume-spread effects of the treatment initialized with cisplatin as seen in Fig. 4a (panel 48 h). After alternating the treatment on day 2, both cisplatin initialized, and Taxol initialized treatments are found to exhibit identical spatial effects (panel 72 h). This shows that irrespective of the initialization strategy, the spatial effects should converge for alternating drug treatments of same duration. However, this result should not be generalized to the overall efficacy of the treatment strategy or the cell viability quantification. The results of cell viability are summarized in Fig. 4c. The experimental data and simulation results show that the average cell viability at the end of treatment (day 4) is lower for Taxol initialized treatment strategies than cisplatin initialized treatments. Thus, although the spatial effects converge with progress of treatment schedule, the efficacy of the treatments is dependent on the order of scheduling.

In contrast to the outcomes of 24-h scheduling, 12-h scheduling produces similar spatial cytotoxic effects and cell viabilities irrespective of cisplatin or Taxol initialization as shown in Fig. 4b and c. An additional phenomenon that occurs in the 12-h scheduling is the drastic reduction in size of the spheroid with progress of time. From the viability plots, we observe that 12-h scheduling is more efficacious than 24-h treatment scheduling, especially for cisplatin-initialized treatment. Hence, the size decrease can be attributed to the expedited cell elimination produced by the 12-h combinatorial treatment. The end points (day 4) of the simulated and experimental viability values for 24-h treatment scheduling, are found to be in a close range, however the model overestimates the effect of cisplatin on day 3. Similarly, the 12-h model simulated viability values follow a trend comparable to the 12-h experimental values. However, they quantitatively differ from each other, with the simulations underestimating the efficacy of the combinatorial treatment. This could arise from the missing numerical parameters for defining any synergistic effects that arise from such short alternation (12 h) durations. Although, 12-h scheduling produced highly efficacious treatment strategy, the use of such scheduling strategy for clinical purposes could be restricted by toxicity and practicality considerations.

### Influence of drugs’ molecular mechanisms on treatment outcomes

Cisplatin through its action of DNA adduct formation causes DNA damage in the cell. If this damage accumulation proceeds unchecked, it results in cell apoptosis. This is the known pathway of cell cycle and cell death progression for cells undergoing apoptosis due to cisplatin penetration. An alternate route of progression would be the repair of the inflicted DNA damage through nucleotide excision [42], proliferating cell nuclear antigen (PCNA) accumulation at damage site [44] and/or mismatch repair (MMR). In the model and experiments, the choice between these two routes is decided by the probability of successful cell repair and the cytotoxic concentration of cisplatin. This is the primary reason for the volume-wide action of cisplatin without any localization effects. On the other hand, paclitaxel works through mitotic spindle stabilization. There is a possibility of mitotic spindle rectification followed by mitosis, or mitotic arrest followed by cell apoptosis. Even though the mechanism of paclitaxel incorporates a stochastic variable, the outcome is not a volume-wide action but rather localized. This is due to its cell cycle specific action which consequently affects only a selective region where this population is localized. Thus, the mechanisms actions, which appear to affect cells randomly, may result in selective population culling based on the primary driving step of drug action. In this study, for cisplatin, the primary is found to be DNA repair, and for paclitaxel, it the mitotic spindle stabilization.

The current model provides a better understanding of how solid tumours are affected by drug treatments. Since a single tumour is comprised of various subclones, some with differing chemosensitivities, targeting the right subpopulation will result in increased drug efficacy. In addition to clonal heterogeneity, spatial distribution of blood vessels also differs within the tumours, such that diffusion gradients vary across the tumour. These can affect the drug response. Tumours may respond to chemotherapy such that only a single smaller tumour focus remain or may respond with multifocal invasive foci scattered across the entire extent of the original tumour. A greater insight into how a tumour will respond has relevance to tumour re-evaluation after neoadjuvant chemotherapy, where it will impact on the decision between a mastectomy or breast conservation surgery.

## Conclusions

In this study, we examined the drug response of human cervical cancer HeLa-C3 cell spheroids to the combinatorial administration of two first line anti-cancer drugs – cisplatin and Taxol. 3D spheroids were employed in our studies instead of 2D cell culture experiments to capture the spatial mass-transfer kinetics and cell growth dynamics. This helps to emulate an environment similar to in-vivo microenvironmental settings. Our mechanistic model was validated by experimental findings that demonstrate the spatio-temporal effects of cisplatin and Taxol. From our studies, it is clear that it is the innate characteristics of Taxol to affect the cells along the periphery of the spheroid. In contrast, the capability of cisplatin is to influence the entire volume of the spheroid limited by the cell DNA repair mechanism. Although, there are multiple studies in literature which analyse the effects of various drug combinations, there is a lack of clear understanding of the localized spatial effects of multiple alternative drug scheduling. In our study, combinatorial scheduling (24 h) of the two drugs was manifested in noticeably different treatment outcomes for different drug ordering. Treatments (24 h) initiated with Taxol were found to be more effective than their counterparts because of the concentric inward penetrating effect of Taxol. The model simulations were validated against experimental data, paving the way towards *in-silico* determination of optimal treatment strategies comprising the drugs and their scheduling. The numerical model enabled us to quantify the effect of such spatial drug activity levels by providing a microscopic picture of the individual cells, their cycle phase, drug uptake, DNA damage, and the other parameters which are difficult to determine in real time using laboratory experiments. Although this numerical model was successfully validated using experiments on spheroids, the model was found to underpredict the treatment outcomes for alternation periods less than 24 h. Such limitations arise from the presence of unknowns such as synergistic effects, which are needed to completely mimic the short-term temporal drug efficacies. Nevertheless, the developed model was capable of capturing single drug-cell interactions and 24-h drug alternation treatments to qualify and quantify the combinatorial drug efficacy. Missing parameters such as hysteresis loops in PK of the drugs and synergy variables could be included in the future models to improve the prediction capability of the simulations. Such *in-silico* models can be used as a test bed for predicting the drug treatment efficacies within practically acceptable accuracies. Improving the model to accommodate for human physiological and tumour micro-environmental parameters will pave way for precise patient specific chemotherapy.

## Data Availability

The datasets used and/or analysed during the current study are available on request.

## Abbreviations

2D: Two dimensional
3D: Three dimensional
C3: Caspase3
CA: Cellular automata
CFP: Cyan Fluorescent protein
DMEM: Dulbecco’s modified Eagle medium
FBS: Fetal bovine serum
FRET: Förster resonance energy transfer
MMR: Mismatch repair
MnSOD: Manganese superoxide dismutase
NER: Nucleotide excision repair
PCNA: Proliferating cell nuclear antigen
PI: Propidium Iodide
PK: Pharmacokinetics
PS: Penicillin/Streptomycin
RFP: Red Fluorescent protein
ROS: Reactive oxygen species
YFP: Yellow Fluorescent protein
